# Patient-reported outcomes of zirconia dental implants: a systematic review and future directions

**DOI:** 10.1186/s41687-025-00839-8

**Published:** 2025-01-14

**Authors:** Behrouz Arefnia, Omid Fakheran, Norbert Jakse, Michael Payer

**Affiliations:** 1 Division of Oral Surgery and Orthodontics, Department of Dental Medicine and Oral Health, Medical University of Graz, Graz, Austria; 2 Division of Restorative Dentistry, Periodontology and Prosthodontics, Department of Dental Medicine and Oral Health, Medical University of Graz, Graz, Austria; 3 Social Dental Medicine Working Group, Department of Dental Medicine and Oral Health, Medical University of Graz, Graz, 8010 Austria

**Keywords:** Dental implants, Zirconia, Patient-reported outcome measures, Systematic review

## Abstract

**Purpose:**

Zirconia dental implants show excellent biocompatibility and tissue integration, low affinity for plaque, and favorable biomechanical properties. However, these objective measures do not adequately replicate the patient’s perception. This systematic review evaluated the evidence on patient-reported outcome (PROs) in zirconia dental implant treatment.

**Methods:**

A systematic literature review was conducted following the PRISMA guidelines, utilizing six electronic databases, and supplemented by a manual search of relevant journals and websites to ensure a thorough and comprehensive screening process. The identified studies were subjected to preidentified inclusion criteria. Only controlled clinical trials published in English were considered without limitations on the year of publication. Data on the study characteristics (follow-up, survival rate (%), implant system, number of implants, and type of treatment), PROMs, level of evidence, and Methodological Index for Nonrandomized Studies (MINORS) Bias Score were extracted.

**Results:**

The initial database and hand search yielded 596 articles; 189 were included in the title and abstract screening after excluding the duplicates. Eighteen articles were selected based on the inclusion criteria, among which six were excluded because they did not match the research question. Thus, the final selection comprised 12 articles. Most PROMs (aesthetics, speaking, comfort, chewing ability, and general satisfaction) at prosthetic delivery revealed significantly improved average scores than those at pretreatment.

**Conclusions:**

Despite the respective limitations of the articles included in this systematic review, patients revealed high satisfaction levels with regard to zirconia dental implants. A high level of heterogeneity was observed among the instruments used for measuring the patient-reported outcomes in patients with zirconia implants, thus highlighting the need to develop specific PROMs in the future.

**Supplementary Information:**

The online version contains supplementary material available at 10.1186/s41687-025-00839-8.

## Introduction

The rehabilitation of completely and partially edentulous patients with dental implants has shown high success due to long-term predictability and satisfactory functioning and aesthetics [1]. Currently, titanium and titanium alloys, the most common materials used for implant manufacturing, have revolutionized dental implantology and raised the standards for tooth replacement [2]. These materials have achieved broad applicability because of their exceptional biocompatibility, favorable mechanical properties, and well-documented positive outcomes [3].

However, despite their acknowledged biological and technological advantages, titanium implants have some significant drawbacks, such as their metallic appearance. The metallic hue can detract from the overall aesthetic appearance, particularly in the aesthetically challenging anterior areas and in patients with a thin gingival biotype [4]. Potential adverse reactions to titanium, such as hypersensitivity, have been reported; however, evidence supporting this assumption is limited [5, 6]. These drawbacks led to the use and exploration of novel materials, such as ceramics, for the production of dental implant fixtures. Due to its exceptional biomechanical qualities, yttria-stabilized tetragonal zirconia is recommended for the manufacture of ceramic dental implants [7, 8]. With regard to biocompatibility and osseointegration, zirconia implants are reported to be comparable to titanium implants [9]. Zirconia implants can be used to avoid significant cosmetic issues following minor peri-implant bone loss or gingival recessions owing to the tooth-like color [10]. Furthermore, several studies have revealed that zirconia ceramics have a lower propensity for bacterial adhesion and biofilm formation, which reduces the risk of peri-implant infection [11, 12]. Another factor supporting zirconia as a ceramic implant material is the desire of certain patients to have metal-free restorations [13].

Several recently published clinical trials and some systematic reviews showed promising clinical performance and a high survival rate for zirconia dental implants [14–16]. However, according to the ‘patient-centered’ care concept, a clinical outcome assessment is insufficient to evaluate the efficacy of an intervention [17–19]. The oral health-related quality of life (OHRQoL) and other psychosocial outcomes of interventions should be considered as essential supplements in clinical trials [20–23]. The term PROMs (Patient-Reported Outcome Measures), which essentially includes “subjective” reports of patients’ perceptions of their oral health status and its impact on their daily life or quality of life, reports of satisfaction with oral health status and/or oral health care, and other nonclinical assessments, was introduced in the eighth European Workshop on Periodontology [24, 25].

To the best of our knowledge, no systematic review has been published on patient-reported outcomes (PROs) related to ceramic dental implants. Therefore, the primary objective of this study was to systematically review the literature to assess the PROs in edentulous patients rehabilitated with zirconia implants. Key outcomes of interest included oral health-related quality of life (OHRQoL), patient satisfaction, and reports on function, aesthetics, and speech. The secondary objective was to summarize and evaluate the tools used to measure PROs in patients receiving zirconia dental implants.

## Materials and methods

### Review of development and focused questions

This systematic review was conducted in accordance with the Cochrane Handbook and reported according to the Preferred Reporting Items for Systematic Reviews and Meta-analyses (PRISMA) statement items [26, 27]. A protocol for the International Prospective Register of Systematic Reviews (PROSPERO) database (ID: CRD42023484023) was developed and submitted.

The PICO question guiding this systematic review is: ‘In patients with missing teeth (P), how do patient-reported outcomes (O), including satisfaction, pain, and quality of life, following the placement of zirconia dental implants (I) compare to pre-treatment levels (C)?’ This question informed the selection of studies that evaluate the impact of zirconia implants on patient-reported outcomes.

### Eligibility criteria

#### Inclusion criteria

The inclusion criteria for this study were as follows: (1) Human clinical studies comprising randomized controlled trials, controlled trials, prospective studies, retrospective studies, and case series; (2) patients with ceramic implants for partial or complete edentulous rehabilitation; (3) patients with fixed and removable implant-supported prostheses for rehabilitation; (4) outcome variables measured using PROMs; (5) a minimum of ten patients; (6) a minimum of one year of follow-up; and (7) no deadline for publication date.

#### Exclusion criteria

The exclusion criteria were as follows: (1) nonclinical and animal studies, commentaries, review articles, and case reports; (2) unavailability of full-text articles; and (5) studies written in a language other than English.

### Sources and search strategy

An electronic literature search for articles published until November 24, 2023, was conducted using various computerized databases, such as the Cochrane Library, MEDLINE, Web of Science, PsycINFO, Scopus, and Google Scholar. The initial literature search contained no restrictions on language or publication date, and the following search terms were used in this systematic review:

((zirconia implant*) OR (ceramic implant*)) AND (((((patient-reported outcome*) OR (patient-related outcome*)) OR (patient satisfaction)) OR (oral health-related quality of life*)).

Phrases and keywords were modified for every database, as required (Supplementary file 1).

To ensure a comprehensive screening process, the electronic search was supplemented by a manual search in the following journals: *Clinical Implant Dentistry and Related Research*, *Journal of Clinical Periodontology*, *Journal of Periodontology*, *Journal of Oral and Maxillofacial Surgery*, *Clinical Oral Implants Research*, *The International Journal of Oral & Maxillofacial Implants*, *International Journal of Oral Implantology*, *Journal of Dental Research*, *Clinical Oral Investigations*, and *International Journal of Periodontics and Restorative Dentistry*. The manual search covered the period from January 1, 2010, to November 24, 2023. Additionally, a thorough manual search of the retrieved articles, their bibliographies, and the following websites was performed: http://clinicaltrials.gov, http://www.centerwatch.com, and http://www.clinicalconnection.com.

### Study selection & data extraction

Two reviewers (OF and MP) independently assessed the titles and abstracts at the initial stage of the study selection. The same reviewers subsequently reviewed and examined the full-text articles [27]. Disagreements were resolved through discussion, and publications that did not meet the eligibility criteria were excluded.

Next, using a piloted, standardized data collection sheet, the data was extracted and assimilated.

According to the objectives of this study, all data were classified according to the year of publication, country, first author, sample characteristics, follow-up time, survival rate, implant system, number of implants, and type of treatment. Furthermore, the instruments used in the included studies were categorized for evaluating the PROs and the related results.

### Quality assessment

Almost all articles included in this systematic review had a nonrandomized study design. Accordingly, the risk of bias and methodological quality were evaluated using the validated Methodological Index for Nonrandomized Studies (MINORS) checklist, which is used to analyze the quality of nonrandomized clinical studies [28]. Each of the 8 or 12 items on the checklist received a score of 0 if the item was not reported, 1 if it was only partially reported, and 2 if it was fully reported. The MINORS score is the sum of the points of the individual items, with a maximum score of 24 for comparative studies (12 items) and 16 points for noncomparative studies (8 items). Version 2 of the Cochrane risk of bias tool for randomized trials (RoB 2) was used for one study, a double-blind randomized controlled clinical trial [29]. In addition, we assigned a level of evidence for each article using the classification system described by Wright et al. [30].

### Results

**Fig. 1:**
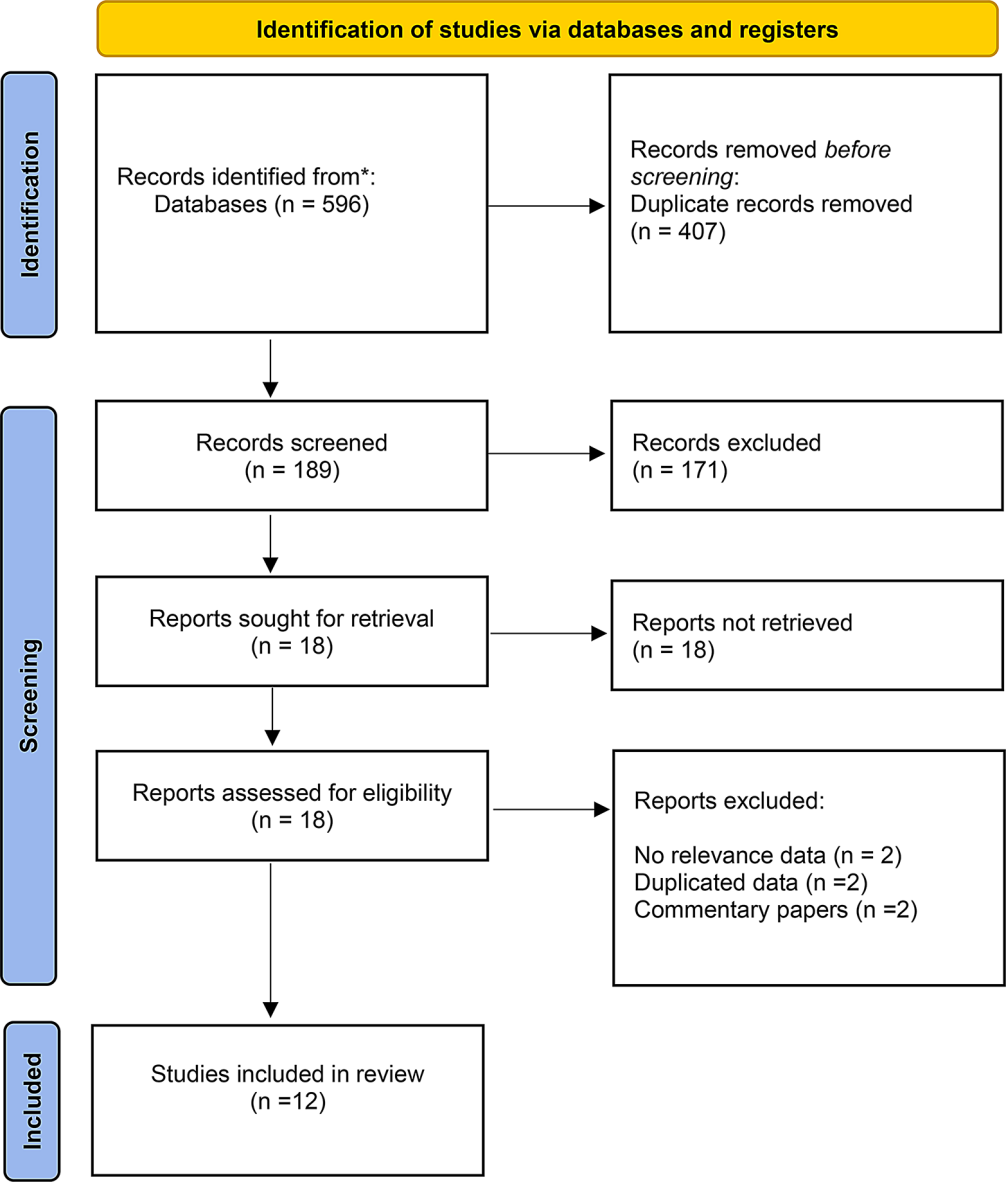
Flow diagram for selection of articles

The electronic database search resulted in 596 publications. After removing the duplicates, 407 titles were excluded, and no additional studies were included after manual searching; consequently, 189 abstracts were examined by the reviewers. With an interexaminer agreement (κ) of 0.89 for abstract selection, 18 papers were selected for full-text examination. Disagreements were settled by discussion or, if needed, by consulting a third researcher before a conclusion was reached. Six articles were eliminated based on the findings of the full-text review phase; these publications were excluded from the reasons listed in the PRISMA selection procedure flow diagram (Fig. 1). Finally, 12 qualified papers were included in the systematic review using predetermined inclusion and exclusion criteria.

Bias analysis was performed using the MINORS criteria for eleven studies that accomplished nonrandomized clinical trials (Supplementary file 2). Accordingly, all studies showed a low risk of bias. Furthermore, the evaluation of one included randomized clinical trial with RoB 2 concluded a low risk of bias (Table 1). Table 2 presents comprehensive details about the characteristics of the articles included in this study.

**Table 1:** Risk of bias assessment according to MINORS and level of evidence

First author Study design	Title	Level of evidence	MINORS bias score
Spies [45] prospective case series	Clinical and Patient-reported Outcomes of a Zirconia Oral Implant: Three-year Results of a Prospective Cohort Investigation	Level IV	12
Holländer [63] Clinical trial	Zirconia Dental Implants: Investigation of Clinical Parameters, Patient Satisfaction, and Microbial Contamination.	Level II	14
Spies [46] Prospective cohort study	Clinical and patient-reported outcomes of zirconia-based implant fixed dental prostheses: Results of a prospective case series 5 years after implant placement.	Level II	15
Spies [47] Prospective cohort study	All-ceramic, bi-layered crowns supported by zirconia implants: Three-year results of a prospective multicenter study	Level II	14
Lorenz [62] Prospective clinical study	Prospective controlled clinical study investigating long-term clinical parameters, patient satisfaction, and microbial contamination of zirconia implants	Level II	12
Spies [48] Prospective cohort study	All-ceramic single crowns supported by zirconia implants: 5‐year results of a prospective multicenter study	Level II	14
Kohal [10] Prospective cohort study	A Prospective Clinical Cohort Investigation on Zirconia Implants: 5-Year Results	Level II	12
Kunavisarut [61] Prospective cohort study	A Pilot Study of Small-Diameter One-Piece Ceramic Implants Placed in Anterior Regions: Clinical and Esthetic Outcomes at 1-Year Follow-up.	Level II	14
Ruiz Henao [33] Randomized clinical trial	Titanium vs. ceramic single dental implants in the anterior maxilla: A 12-month randomized clinical trial	Level I	Low risk of bias
Sala [31] Retrospective case series study	Clinical evaluation and patient related outcomes of one- and two-piece zirconia implants at five years of loading: A case series study	Level IV	11
Rutkowski [34] Retrospective clinical study	Success and patient satisfaction of immediately loaded zirconia implants with fixed restorations one year after loading	Level III	11
Kohal [32] Prospective cohort study	One-Piece Zirconia Oral Implants for Single Tooth Replacement: Five-Year Results from a Prospective Cohort Study	Level II	13

**Table 2:** Study characteristics of included studies

Study/Year Country	Sample	Follow-up	Survival rate (%)	Implant system	Number of implants and Type of treatment
Spies 2015 Germany [45]	40 patients: 20 women 20 men	36 months	94.2% after 3 y	ATZ Ziraldent implants	Total: 53 ceramic implants 51 implants: Healed sites 2 implants: Fresh extraction sites Single implant or bridge restorations.
Holländer 2016 Germany [63]	38 Patients: 20 women 18 men Age: 56.24 ± 10.6 Y Range: 33 to 74 Y	12 months	100%	(Z-Systems, Oensingen, Switzerland)	106 implants in different regions of the Maxilla and the Mandible. + All-ceramic superstructure
Spies 2017 a Germany [46]	13 patients: 6 women 7 men Age: 41–78 Y	60 months	100%	ATZ Ziraldent implants	Not reported
Spies 2017 b Germany/Switzerland [47]	44 patients: 19 women 25 men Age: 46.6 ± 13.1 Y Range: 25–69 Y	36 Months	100%	Vitaclinical, VITA Zahnfabrik; Bad Säckingen, Germany	44 posterior Implants with single-tooth restoration, located in the region of a former premolar (*n* = 17) or molar (*n* = 27)
Lorenz 2019 Germany [62]	28 patients: 15 women 13 men Age: 63.5 Y Range 39–80 Y	93 months	100%	(Z-Systems, Oensingen, Switzerland)	83 zirconia implants 38 in maxilla. 45 in mandible.
Spies 2019 Germany/Switzerland [48]	44 patients 19 women, 25 men Age: 46.6 ± 13.1 Y Range: 25–69 Y	61.0 ± 1.4 months	97.5 ± 2.47%.	Vitaclinical, VITA Zahnfabrik; Bad Säckingen, Germany	44 posterior Implants with single-tooth restoration. Located in the region of a former premolar (*n* = 17) or molar (*n* = 27).
Kohal 2020 Germany [10]	40 patients Age range: 18–70 Y	60 months	94.3%	(ATZ) implant (Metoxit AG, Thayngen, Switzerland)	27 single crown implants, and 26 implants for 3-unit FDP.
Kunavisarut 2020 Thailand [61]	20 patients 14 women, 6 men Age: 52.60 ± 12.43 Age range: 25–72	12 months	100%	Straumann PURE Ceramic implants; Narrow Diameter^®^; Switzerland	20 single anterior implants in maxilla (*n* = 15) and mandible (*N* = 5). 3.3 mm zirconia implants.
Ruiz Henao 2021 Spain [33]	30 patients 16 women, 14 men Age: 54.13	12 months	100%	Straumann PURE Ceramic implants; Narrow Diameter^®^; Switzerland	30 single anterior implants in maxilla (16 Ceramic implants and 14 titanium implants)
Sala 2022 Spain [31]	18 patients: 10 women, 8 men Age: 52 ± 9.1 Y.	54.92 ± 7.3 months	86%	The Ceralog implant system (Camlog Biotechnologies AG, Basel, Switzerland)	29 Single or multiple crowns implants.
Rutkowski 2022 Germany [34]	41 patients: Mean age: 57 Y PROMs sample size: *N* = 22 (53.7)	21 months	92%	Swiss Dental Solutions AG, Kreuzlingen, Switzerland.	163 immediately or conventionally placed one-piece and two-piece zirconium dioxide implants in different anatomical regions of the maxilla and mandible
Kohal 2023 Germany [32]	65 patients Age range: 18–70 Y	60 months	78.2%	Nobel Biocare AB, Gothenburg, Sweden).	66 single-tooth implants

The trials in the articles included were performed between 2015 and 2023. The majority of the studies were conducted in Germany (*n* = 7), two were conducted in Spain, and one in Thailand. The studies evaluated 13 to 65 dental implant patients, mostly middle-aged, with an age range of 18 to 80 years and a roughly equal distribution of sex. The minimum follow-up period among the studies was 12 months, and the maximum was 93 months. Most studies (*n* = 10) showed a high survival rate for ceramic implants within the follow-up period (92–100%). A survival rate of 86% was reported by Sala et al. after 54.92 ± 7.3 months [31]. The lowest survival rate (78.2%) was reported by Kohal et al. in Germany after a 60-month investigation [32].

Almost all the included studies (*n* = 11) used researcher-made questionnaires to evaluate the PROs. Various patient-centered outcomes, including aesthetics, chewing ability, speech, comfort, self-esteem, and general satisfaction, were investigated in these studies (Table 3). The findings indicated that patient satisfaction with all PROs increased significantly with all measurements. However, a meta-analysis could not be conducted due to the marked heterogeneity among the instruments used for evaluating the PROs in the included studies; instead, a descriptive data synthesis was performed. Accordingly, eight studies used visual analog scales of different sizes and scoring protocols to measure the perceptions of the patients with regard to the outcomes. Moreover, five studies implemented numeric questionnaires with various grading systems to collect the patients’ opinions (Table 3). The outcomes between the ceramic and titanium implant groups were compared in one study only, and no significant differences in PROMs were reported between the two groups [33].

**Table 3:** PROMs and PROs of included studies

Study/Year	Type of PROM	Target of measure and results
Spies 2015 [45]	A Researcher-made questionnaire: Visual Analogue Scales (VAS).	All follow-up assessments revealed significantly improved average VAS values at the delivery of the prosthetic restorations (81–97.7%; *P* < 0.038) compared with the pretreatment situation (33.9-85.1%). The improvement of sense and self-esteem remained stable over the course of the follow-ups (*P* = 0.128). Subjective patients’ perceptions of function, aesthetics, and speech still increased over time (*P* < 0.022).
Holländer 2016 [63]	A Researcher-made questionnaire: Consisting of 6-grade scale ranging from positive (Grade 1: very good) to negative (Grade 6:Unsatisfactory)	Comfort: 1.34 ± 0.53 Esthetics: 1.34 ± 0.58 Overall results: 1.37 ± 0.49 Acceptance of the treatment: 97.40% Recommend this treatment method: 100%
Spies 2017a [46]	A Researcher-made questionnaire: Visual Analogue Scales (VAS).	Except for the appraisal of speech (88.5%, *p* = 0.341), patients gave significantly higher VAS scores in the remaining four categories (66.0–92.5%, *p* ≤ 0.038). Over the course of the follow-ups the initially improved perception of function (eating), esthetic/appearance and self-esteem remained stable (*p* ≥ 0.057). The perception of sense (*p* = 0.030) and speech (*p* = 0.012) increased over time.
Spies 2017b [47]	A Researcher-made questionnaire: Visual Analogue Scales (VAS).	All follow-up assessments revealed significantly improved average VAS (function: +18.3%, esthetic: +22.2%, self-esteem: +15.6%; *p* < 0.001) except the appraisal of speech (+ 0.8%; *p* = 0.139) These improvements remained unaffected until the end of the 3-year follow-up (85.4–92.7%, *p* > 0.390).
Lorenz 2019 [62]	A Researcher-made questionnaire: Consisting of 10-grade scale ranging from positive (Grade 1: very good) to negative (Grade 10: Unsatisfactory).	All questions (patient´s satisfaction concerning the surgical intervention, the management, esthetics, and the overall patient satisfaction with the treatment method) revealed a point score between 1 and 2 with a mean point score of 1.3. None of the patients mentioned a foreign-body feeling, all patients mentioned high confidence in the zirconia material and would choose zirconia implants again as the treatment of choice.
Spies 2019 [48]	A Researcher-made questionnaire: Visual Analogue Scales (0-100).	All PROMs at prosthetic delivery except for speech (*p* = 0.139) showed significantly improved VAS scores (81–94%; *p* < 0.001). No decrease in satisfaction could be observed over time until the 5- year follow‐up (93–97%).
Kohal 2020 [10]	Researcher-made questionnaire: Visual Analogue Scales (0-100).	All follow-up assessments revealed significantly improved average VAS values at the delivery of the prosthetic restorations (81–93.5%) Compared with the pretreatment situation (33.9–85.2%). The improvement of function, speech, and self-esteem remained stable over the course of the follow-ups. Subjective patients’ perceptions of esthetics and sense significantly increased over time.
Kunavisarut 2020 [61]	A Researcher-made questionnaire: Visual Analogue Scales (0-100).	Overall satisfaction: 93.3 ± 7.8 Speech function:95.1 ± 5.3 Masticatory function: 93.6 ± 7.6 Esthetics: 94.5 ± 6.2
Ruiz Henao 2021 [33]	A ten-grade numeric questionnaire (0–10) to assess patients’ satisfaction.	All PROMs (Esthetics, Speaking, Comfort, Chewing ability and General satisfaction) at prosthetic delivery revealed significantly improved average scores compared with the pretreatment situation in ceramic and titanium implant groups. There were no significant differences between ceramic and titanium implant groups with respect to PROMs.
Sala 2022 [31]	A six-point ordinal scale (Grade 1: Unsatisfactory, Grade 6: very good) for assessing patients’ satisfaction. A Visual analog scale (0–10) for evaluating satisfaction with the esthetic outcome.	Questionnaire and VAS results both demonstrated a good level of satisfaction with the treatment. Patient-related outcomes were as follows: Chewing comfort score: 4.93 ± 0.27 Phonetic ability score: 5.86 ± 0.38 Chewing comfort (vs. natural teeth): 4.86 ± 0.36 Implant reconstruction cleaning score: 4.86 + 1.35 Overall patient expectation fulfillment score: 5.43 ± 1.2. The mean VAS score for evaluating satisfaction with the esthetic: 8.8
Rutkowski 2022 [34]	A modified OHIP Edent (Oral Health Impact Profile) questionnaire	An average overall score of 0.54/100 points (maximum score 100, low score = favorable satisfaction) was obtained for the patient survey, suggesting high patient satisfaction.
Kohal 2023 [32]	A Researcher-made questionnaire: Visual Analogue Scales (0-100).	All PROMs assessments revealed improvements of the average VAS values (function: from 72.2 to 91.8; aesthetics: from 63.5 to 92.4; sense: from 36.6 to 88.5; speech: from 90.0 to 94.4; self-esteem: from 75.6 to 91.2) compared to the pre-treatment situation (36.6–90.0%).

Rutkowski et al. used a modified version of the oral health impact profile questionnaire called the OHIP Edent, wherein the maximum score is 100, and lower scores indicate greater satisfaction; the authors reported an average score of 0.54 for ceramic implant recipients, suggesting high patient satisfaction [34].

## Discussion

This systematic review summarizes current evidence on the PROs and satisfaction with zirconia implant treatment. In contrast to previous systematic reviews that focused on clinical and radiographic outcomes, this review is the first to focus on patients’ perspectives regarding ceramic dental implants [14, 15, 35, 36].

There is conflicting data on the survival rates of zirconia [15, 37, 38]. Nevertheless, a recent meta-analysis estimated a survival rate of 97.2% (confidence interval [CI], 94.7–99.1) in five years for zirconia implants [14]. The rehabilitation of partially edentulous patients with zirconia implants showed a comparable survival and success rate to titanium implants on a short-term follow-up, hence, it could be an approach of choice, especially in the aesthetic zones [8, 39, 40].

Ceramic implants may play a more prominent role in the care of edentulous patients in the future [41]. Therefore, considering the experiences of patients treated with zirconia implants is essential for enhancing the quality of rehabilitation. A user-centered approach can improve patient satisfaction and acceptance of the treatment [42]. Furthermore, the patients’ perspectives will ultimately help in meeting their expectations more accurately [43]. According to the results of the current study, patients revealed a high level of satisfaction with regard to function, aesthetics, comfort, and self-esteem following treatment with ceramic dental implants, even after five years. However, this result should be taken cautiously owing to the few studies available, the combination of several implant systems, and various implant sites. Notably, almost all investigations had a nonrandomized design. Randomized controlled studies must be conducted to compare the PROs of ceramic and titanium implants.

The current systematic review also highlighted the substantial shortcomings in the instruments available for measuring PROs in implant dentistry. Most researchers used questionnaires they had created themselves, which were unvalidated and highly diverse. These questionnaires consisted of items that were important for the oral health-related quality of life according to the viewpoint of the clinicians. Generally, these scales reflect the clinicians’ perspectives on how patients “should feel” rather than what patients “really feel.” The essence of PROMs is to explore the perceived outcomes that come directly from the patient without interpretation or restriction by a clinician or anyone else [44]. Nevertheless, when investigators use researcher-made questionnaires, the PROs are restricted to the physicians’ perspective, and patients cannot add any additional outcomes to these predetermined options. In addition to these concerns, it is important to note that 6 out of the 12 studies included in this review were authored by the same two researchers, Spies and Kohal [10, 31, 32, 45–48]. This may have influenced the selection and use of specific PRO measures in these studies, potentially introducing bias in the findings.

Despite clinicians’ intentions, evidence-based decision-making, and technical competence, a discrepancy between the dentist’s definition of a successful implant treatment and the patient’s perception is not unusual [49].

Albrektsson et al. defined that a successful implant must present with a bone loss of < 0.2 mm per year after the first year of loading and no mobility, peri-implant radiolucency, persistent pain, discomfort, or infection [50]. However, patients’ expectations regarding dental implant therapy are not limited to these objective indicators. According to the results of qualitative studies, patients expected implants to overcome not only their functional problems but also the social and psychological difficulties they experienced with missing teeth or conventional dentures [51, 52]. The patients expected implants to restore their “normal” appearance and enable them to feel more confident and relaxed in social interactions, an expectation that has also been reported among patients seeking veneers [53]. According to the results of this comprehensive literature review and the conclusions of other systematic reviews and international consensus reports regarding PROMs in dental implants, a specific PROM for dental implant patients is currently unavailable [17, 54]. Thus, the available PROMs must be improved to implement person-centered care in implant therapy [55, 56].

In addition, the inherent distinctions between real world and controlled clinical study environments should be considered when discussing PROs. Researchers make every effort to meet al.l the demands of the patients during and after the treatment period to retain them in the study and assess the long-term outcomes. This special attention naturally leads to a high degree of satisfaction, generally in line with the tenets of person-centered dentistry [57]. In contrast, patients in the real world may not receive the attention they desire for various reasons, including time constraints and a lack of intersectoral coordination, which might lead to lower patient satisfaction [58]. Thus, exploring the lived experience of recipients of ceramic dental implants in the long-term should be considered to fully understand the patient’s expectations and the desired outcomes in the real world.

Evidence of discrepant views between patients and dentists in their assessment of dental implant treatment outcomes highlights the irrefutable need for simple and effective methods to enhance patient involvement in developing and evaluating PROMs.

To ensure that patient-centered instruments for dental implants are relevant, reliable, and valid, future research should adhere to established guidelines, including those from the Scientific Advisory Committee of the Medical Outcomes Trust and the U.S. Food and Drug Administration, to develop specific PROMs for dental implant patients [59, 60]. These guidelines emphasize a rigorous process that involves concept elicitation, item generation, and cognitive debriefing, all of which are essential to ensuring that PROMs accurately reflect patient experiences and outcomes. Psychometric testing, as the final stage of this process, is crucial for confirming the reliability and validity of PROMs, ensuring that they measure what they intend to and yield consistent results. Mixed-methods studies, which combine both quantitative and qualitative approaches, are particularly valuable in this context. By following these established methodologies, future research can generate robust, patient-centered evidence that will inform clinical decision-making and ultimately enhance patient care in the field of dental implants.

## Conclusions

Given the various limitations of the articles included in this systematic review, patients reported high levels of satisfaction with zirconia dental implants. The instruments used to measure patient-reported outcomes in patients with zirconia implants showed a high degree of heterogeneity, highlighting the need for future development of specific PROMs.

## Electronic supplementary material

Below is the link to the electronic supplementary material.

Supplementary Material 1

Supplementary Material 2

## Data Availability

The datasets used and/or analysed during the current study available from the corresponding author on reasonable request.

## Abbreviations

OHIP: Oral health impact profile
PICO: Population, intervention, comparison, and outcome
PRISMA: Preferred Reporting Items for Systematic Reviews and Meta-analyses
PRO: Patient-reported outcomes
PROM: Patient-reported outcome measures

## References

[1] Duong HY et al (2022) Oral health-related quality of life of patients rehabilitated with fixed and removable implant-supported dental prostheses. Periodontol 2000 88(1):201–237 10.1111/prd.12419 PMC9304161 35103325

[2] Hoque ME et al (2022) Titanium and titanium alloys in dentistry: current trends, recent developments, and future prospects. Heliyon 8(11):e11300 36387463 10.1016/j.heliyon.2022.e11300 PMC9640965

[3] Bosshardt DD, Chappuis V, Buser D (2017) Osseointegration of titanium, titanium alloy and zirconia dental implants: current knowledge and open questions. Periodontol 2000 73(1):22–40 10.1111/prd.12179 28000277

[4] Ramanauskaite A, Sader R (2022) Esthetic complications in implant dentistry. Periodontology 2000 88(1):73–85 10.1111/prd.12412 35103323

[5] Kim KT et al (2019) General review of titanium toxicity. Int J Implant Dentistry 5(1):1–12 10.1186/s40729-019-0162-x PMC6409289 30854575

[6] Poli PP et al (2021) Titanium allergy caused by dental implants: a systematic literature review and case report. Materials 14(18):5239 34576463 10.3390/ma14185239 PMC8465040

[7] Benic GI et al (2017) Guided bone regeneration at zirconia and titanium dental implants: a pilot histological investigation. Clin Oral Implants Res 28(12):1592–1599 28653343 10.1111/clr.13030

[8] Balmer M et al (2022) EAO position paper: current level of evidence regarding Zirconia implants in clinical trials. Int J Prosthodont 35(4):560–566 36125878 10.11607/ijp.8131

[9] Remísio M et al (2023) Histologic osseointegration level comparing Titanium and Zirconia Dental implants: Meta-analysis of Preclinical studies. Int J Oral Maxillofac Implants 38(4):667–680 37669522 10.11607/jomi.10142

[10] Kohal RJ et al (2020) A prospective clinical Cohort Investigation on Zirconia implants: 5-Year results. J Clin Med 9(8):2585 32785031 10.3390/jcm9082585 PMC7464596

[11] Al-Radha ASD et al (2012) Surface properties of titanium and zirconia dental implant materials and their effect on bacterial adhesion. J Dent 40(2):146–153 22182466 10.1016/j.jdent.2011.12.006

[12] Rimondini L et al (2002) Bacterial colonization of zirconia ceramic surfaces: an in vitro and in vivo study. Int J Oral Maxillofac Implants 17(6):793–798 12507238

[13] Han J, Zhao J, Shen Z (2017) Zirconia ceramics in metal-free implant dentistry. Adv Appl Ceram 116(3):138–150

[14] Roehling S et al (2023) Clinical and radiographic outcomes of zirconia dental implants-A systematic review and meta-analysis. Clin Oral Implants Res 34(Suppl 26):112–124 37750521 10.1111/clr.14133

[15] Padhye NM et al (2023) Survival and success of zirconia compared with titanium implants: a systematic review and meta-analysis. Clin Oral Invest 27(11):6279–6290 10.1007/s00784-023-05242-5 PMC10630218 37740825

[16] Karapataki S et al (2023) Clinical performance of two-piece zirconia dental implants after 5 and up to 12 years. Int J Oral Maxillofac Implants 38(6):1105–1114 10.11607/jomi.10284 38085741

[17] Feine J et al (2018) Group 3 ITI consensus report: patient-reported outcome measures associated with implant dentistry. Clin Oral Implants Res 29:270–275 30328187 10.1111/clr.13299

[18] Scambler S, Asimakopoulou K (2014) A model of patient-centred care–turning good care into patient-centred care. Br Dent J 217(5):225–228 25213518 10.1038/sj.bdj.2014.755

[19] Tsoi JK, Ding H (2023) A narrative review on the overlooked aspects of dPROs in connection with dental materials. J Evid Based Dent Pract 23(1s):101796 36707171 10.1016/j.jebdp.2022.101796

[20] Mercieca-Bebber R et al (2018) The importance of patient-reported outcomes in clinical trials and strategies for future optimization. Patient Relat Outcome Meas 9:353–367 10.2147/PROM.S156279 PMC6219423 30464666

[21] Parhizkar P et al (2022) Can adjunctive corticosteroid therapy improve patient-centered outcomes following third molar surgery? A systematic review. Med Oral Patol Oral Cir Bucal 27(5):e410 10.4317/medoral.25177 PMC9445603 35975802

[22] Duong HY et al (2022) Oral health-related quality of life of patients rehabilitated with fixed and removable implant‐supported dental prostheses. Periodontol 2000 88(1):201–237 35103325 10.1111/prd.12419 PMC9304161

[23] Yu X et al (2023) A bibliometric mapping study of the literature on oral health-related quality of life. J Evid Based Dent Pract 23(1s):101780 36707159 10.1016/j.jebdp.2022.101780

[24] Phonsuda Chanthavisouk RDH (2022) Dental therapy and dental patient-reported outcomes (dPROs). J Evidence-Based Dent Pract 22(1):101660 10.1016/j.jebdp.2021.101660 35063179

[25] Lang NP, Zitzmann NU, Working Group 3 of the VIII European Workshop on Periodontology (2012) Clinical research in implant dentistry: evaluation of implant-supported restorations, aesthetic and patient‐reported outcomes. J Clin Periodontol 39:133–138 22533953 10.1111/j.1600-051X.2011.01842.x

[26] Cumpston M et al (2019) Updated guidance for trusted systematic reviews: a new edition of the Cochrane Handbook for Systematic Reviews of Interventions. Cochrane Database Syst Rev. 2019(10) 10.1002/14651858.ED000142 PMC10284251 31643080

[27] Page MJ et al (2021) The PRISMA 2020 statement: an updated guideline for reporting systematic reviews. Int J Surg 88:105906 33789826 10.1016/j.ijsu.2021.105906

[28] Slim K et al (2003) Methodological index for non-randomized studies (MINORS): development and validation of a new instrument. ANZ J Surg 73(9):712–716 12956787 10.1046/j.1445-2197.2003.02748.x

[29] Sterne JA et al (2019) RoB 2: a revised tool for assessing risk of bias in randomised trials. BMJ 366:l4898 10.1136/bmj.l4898 31462531

[30] Wright JG, Swiontkowski MF, Heckman JD (2003) Introducing levels of evidence to the journal. J Bone Joint Surg Am 85(1):1–3 12533564

[31] Sala L et al (2023) Clinical evaluation and patient related outcomes of one-and two‐piece zirconia implants at five years of loading: a case series study. J Esthetic Restor Dentistry 35(4):577–585 10.1111/jerd.13002 36583946

[32] Kohal R-J et al (2023) One-piece zirconia oral implants for single tooth replacement: five-year results from a prospective cohort study. J Funct Biomaterials 14(2):116 10.3390/jfb14020116 PMC9964460 36826915

[33] Ruiz Henao PA et al (2021) Titanium vs ceramic single dental implants in the anterior maxilla: a 12-month randomized clinical trial. Clin Oral Implants Res 32(8):951–961 10.1111/clr.13788 34061402

[34] Rutkowski R et al (2022) Success and patient satisfaction of immediately loaded zirconia implants with fixed restorations one year after loading. BMC Oral Health 22(1):198 35606734 10.1186/s12903-022-02231-0 PMC9125844

[35] Haimov E et al (2023) Differences in Titanium, Titanium-Zirconium, Zirconia implants Treatment outcomes: a systematic literature review and Meta-analysis. J Oral Maxillofac Res 14(3):e1 37969951 10.5037/jomr.2023.14301 PMC10645476

[36] Mohseni P, Soufi A, Chrcanovic BR (2023) Clinical outcomes of zirconia implants: a systematic review and meta-analysis. Clin Oral Investig 28(1):15 38135804 10.1007/s00784-023-05401-8 PMC10746607

[37] Afrashtehfar KI, Fabbro MD (2020) Clinical performance of zirconia implants: a meta-review. J Prosthet Dent 123(3):419–426 31451193 10.1016/j.prosdent.2019.05.017

[38] Koller M et al (2020) Two-piece zirconia versus titanium implants after 80 months: clinical outcomes from a prospective randomized pilot trial. Clin Oral Implants Res 31(4):388–396 31944420 10.1111/clr.13576

[39] Karapataki S et al (2023) Clinical performance of two-piece Zirconia Dental implants after 5 and up to 12 years. Int J Oral Maxillofac Implants 38(6):1105–1114 38085741 10.11607/jomi.10284

[40] Steyer E et al (2021) Immediate Restoration of single-piece Zirconia implants: a prospective Case Series—Long-Term results after 11 years of clinical function. Materials 14(22):6738 34832139 10.3390/ma14226738 PMC8621133

[41] Neugebauer J et al (2023) Ceramic Dental implants: a systematic review and Meta-analysis. Int J Oral Maxillofac Implants 38(suppl):30–36 37436947 10.11607/jomi.10500

[42] Hanson K et al (2022) The Lancet Global Health Commission on financing primary health care: putting people at the centre. Lancet Global Health 10(5):e715–e772 35390342 10.1016/S2214-109X(22)00005-5 PMC9005653

[43] Grover S et al (2022) Defining and implementing patient-centered care: an umbrella review. Patient Educ Couns 105(7):1679–1688 34848112 10.1016/j.pec.2021.11.004

[44] Costa DS et al (2021) How is quality of life defined and assessed in published research? Qual Life Res 30:2109–2121 33792834 10.1007/s11136-021-02826-0

[45] Spies B et al (2015) Clinical and patient-reported outcomes of a zirconia oral implant: three-year results of a prospective cohort investigation. J Dent Res 94(10):1385–1391 26232388 10.1177/0022034515598962

[46] Spies BC et al (2018) Clinical and patient-reported outcomes of zirconia‐based implant fixed dental prostheses: results of a prospective case series 5 years after implant placement. Clin Oral Implants Res 29(1):91–99 28940708 10.1111/clr.13072

[47] Spies BC et al (2017) All-ceramic, bi-layered crowns supported by zirconia implants: three-year results of a prospective multicenter study. J Dent 67:58–65 28939484 10.1016/j.jdent.2017.09.008

[48] Spies BC et al (2019) All-ceramic single crowns supported by zirconia implants: 5‐year results of a prospective multicenter study. Clin Oral Implants Res 30(5):466–475 30972828 10.1111/clr.13433

[49] Abrahamsson KH et al (2017) Altered expectations on dental implant therapy; views of patients referred for treatment of peri-implantitis. Clin Oral Implants Res 28(4):437–442 26918305 10.1111/clr.12817

[50] Albrektsson T et al (1986) The long-term efficacy of currently used dental implants: a review and proposed criteria of success. Int J Oral Maxillofac Implants 1(1):11–25 3527955

[51] Kashbour W et al (2015) Patients’ experiences of dental implant treatment: a literature review of key qualitative studies. J Dent 43(7):789–797 25921332 10.1016/j.jdent.2015.04.008

[52] Malmqvist S et al (2023) Patient’s experiences of dental implants, peri-implantitis and its treatment‐a qualitative interview study. Int J Dent Hyg 22(3):530–539 10.1111/idh.12683 37093891

[53] Grey EB et al (2013) A qualitative study of patients’ motivations and expectations for dental implants. Br Dent J 214(1):E1–E1 23306517 10.1038/sj.bdj.2012.1178

[54] Donos N et al (2021) Impact of timing of dental implant placement and loading: summary and consensus statements of group 1—the 6th EAO consensus conference 2021. Clin Oral Implants Res 32:p85–92 10.1111/clr.13809 34642977

[55] Tzelepis F et al (2015) Measuring the quality of patient-centered care: why patient-reported measures are critical to reliable assessment. Patient Prefer Adherence 9:831–835 10.2147/PPA.S81975 PMC4484696 26150703

[56] Schierz O, Reissmann DR (2021) Dental patient-reported outcomes–the promise of dental implants. Elsevier, p 101541 10.1016/j.jebdp.2021.101541 34051958

[57] Apelian N et al (2017) How can we provide person-centred dental care? Br Dent J 223(6):419–424 28937118 10.1038/sj.bdj.2017.806

[58] Vennedey V et al (2020) Patients’ perspectives of facilitators and barriers to patient-centred care: insights from qualitative patient interviews. BMJ open 10(5):e033449 32376748 10.1136/bmjopen-2019-033449 PMC7223019

[59] Lohr KN (2002) Assessing health status and quality-of-life instruments: attributes and review criteria. Qual Life Res 11:193–205 12074258 10.1023/a:1015291021312

[60] Food U, Administration D (2009) Guidance for industry: patient-reported outcome measures: use in medical product development to support labeling claims. Fed Regist 65132 10.1186/1477-7525-4-79 PMC1629006 17034633

[61] Kunavisarut C, Buranajanyakul L, Kitisubkanchana J, Pumpaluk P (2020) A pilot study of small-diameter one-piece ceramic implants placed in Anterior regions: clinical and esthetic outcomes at 1-year follow-up. Int J Oral Maxillofacial Implants. 35(5). 10.11607/jomi.8308 10.11607/jomi.8308 32991647

[62] Lorenz J, Giulini N, Hölscher W, Schwiertz A, Schwarz F, Sader R (2019) Prospective controlled clinical study investigating long-term clinical parameters, patient satisfaction, and microbial contamination of zirconia implants. Clin Implant Dent Relat Res 21(2):263–271. Epub 2019 Feb 4. PMID: 30714303. 10.1111/cid.12720 10.1111/cid.12720 30714303

[63] Holländer J, Lorenz J, Stübinger S, Hölscher W, Heidemann D, Ghanaati S, Sader R (2016) Zirconia dental implants: investigation of clinical parameters, patient satisfaction, and microbial contamination. Int J Oral Maxillofac Implants 31(4):855–864. PMID: 27447153. 10.11607/jomi.4511 10.11607/jomi.4511 27447153

