# Supplementary material for: Patient-reported outcomes of zirconia dental implants: a systematic review and future directions
Source: J Patient Rep Outcomes. 2025 Jan 14;9:7. doi: 10.1186/s41687-025-00839-8 (PMC11732800; doi:10.1186/s41687-025-00839-8)
Supplement: Supplementary file 1 — Supplementary Material 1 [file 41687_2025_839_MOESM1_ESM.docx]

Supplementary file 1: Electronic Databases and Search Strategies According to the PICO Question Components

**Search date:** November 24, 2023

**MEDLINE (via PubMed)**

(P) #1 ("Dental implants" [MeSH] OR "dental implantation" [MeSH]) AND ("zirconium oxide" [MeSH] OR "yttria-stabilized tetragonal zirconia" [MeSH] OR "zirconia" OR "zirconia implant*" OR "ceramic implant*") (I) #2 ("Quality of Life" OR Quality-of-Life OR OHRQoL OR QoL OR HRQoL OR OHIP OR "Life Quality" OR satisfaction OR dissatisfaction OR "patient preference*" OR "patient outcome*" OR "patient expectation*" OR "treatment outcome*" OR "outcome assessment*" OR "oral health*" OR PROM* OR "patient related outcome*" OR "patient centered outcome*" OR "patient reported outcome*" OR "patient centered care" OR questionnaire* OR interview* OR survey*) #1 AND #2

**Web of Science**

(P) #1 ("Dental implants" OR "dental implantation" OR "zirconium oxide" OR "yttria-stabilized tetragonal zirconia" OR "zirconia" OR "zirconia implant*" OR "ceramic implant*") (I) #2 ("Quality of Life" OR Quality-of-Life OR OHRQoL OR QoL OR HRQoL OR OHIP OR "Life Quality" OR satisfaction OR dissatisfaction OR "patient preference*" OR "patient outcome*" OR "patient expectation*" OR "treatment outcome*" OR "outcome assessment*" OR "oral health*" OR PROM* OR "patient related outcome*" OR "patient centered outcome*" OR "patient reported outcome*" OR "patient centered care" OR questionnaire* OR interview* OR survey*) #1 AND #2

**PsycINFO**

(P) #1 ("Dental implants" OR "dental implantation" OR "zirconium oxide" OR "yttria-stabilized tetragonal zirconia" OR "zirconia" OR "zirconia implant*" OR "ceramic implant*") (I) #2 ("Quality of Life" OR Quality-of-Life OR OHRQoL OR QoL OR HRQoL OR OHIP OR "Life Quality" OR satisfaction OR dissatisfaction OR "patient preference*" OR "patient outcome*" OR "patient expectation*" OR "treatment outcome*" OR "outcome assessment*" OR "oral health*" OR PROM* OR "patient related outcome*" OR "patient centered outcome*" OR "patient reported outcome*" OR "patient centered care" OR questionnaire* OR interview* OR survey*) #1 AND #2

**Cochrane Library**

(P) #1 ("Dental implants" OR "dental implantation") #2 ("zirconium oxide" OR "yttria-stabilized tetragonal zirconia" OR "zirconia" OR "zirconia implant*" OR "ceramic implant*") (I) #3 ("Quality of Life" OR Quality-of-Life OR OHRQoL OR QoL OR HRQoL OR OHIP OR "Life Quality" OR satisfaction OR dissatisfaction OR "patient preference*" OR "patient outcome*" OR "patient expectation*" OR "treatment outcome*" OR "outcome assessment*" OR "oral health*" OR PROM* OR "patient related outcome*" OR "patient centered outcome*" OR "patient reported outcome*" OR "patient centered care" OR questionnaire* OR interview* OR survey*) #1 AND #2 AND #3

**Scopus**

(P) #1 ("Dental implants" OR "dental implantation" OR "zirconium oxide" OR "yttria-stabilized tetragonal zirconia" OR "zirconia" OR "zirconia implant*" OR "ceramic implant*") (I) #2 ("Quality of Life" OR Quality-of-Life OR OHRQoL OR QoL OR HRQoL OR OHIP OR "Life Quality" OR satisfaction OR dissatisfaction OR "patient preference*" OR "patient outcome*" OR "patient expectation*" OR "treatment outcome*" OR "outcome assessment*" OR "oral health*" OR PROM* OR "patient related outcome*" OR "patient centered outcome*" OR "patient reported outcome*" OR "patient centered care" OR questionnaire* OR interview* OR survey*) #1 AND #2

### ****Google Scholar****

**Search**: "Dental implants" OR "dental implantation" OR "zirconium oxide" OR "yttria-stabilized tetragonal zirconia" OR "zirconia" OR "zirconia implant*" OR "ceramic implant*" AND ("Quality of Life" OR Quality-of-Life OR OHRQoL OR QoL OR HRQoL OR OHIP OR "Life Quality" OR satisfaction OR dissatisfaction OR "patient preference*" OR "patient outcome*" OR "patient expectation*" OR "treatment outcome*" OR "outcome assessment*" OR "oral health*" OR PROM* OR "patient related outcome*" OR "patient centered outcome*" OR "patient reported outcome*" OR "patient centered care" OR questionnaire* OR interview* OR survey*)
