# Supplementary material for: Patient-reported outcomes of zirconia dental implants: a systematic review and future directions
Source: J Patient Rep Outcomes. 2025 Jan 14;9:7. doi: 10.1186/s41687-025-00839-8 (PMC11732800; doi:10.1186/s41687-025-00839-8)
Supplement: Supplementary file 2 — Supplementary Material 2 [file 41687_2025_839_MOESM2_ESM.docx]

**Supplementary table 2.** Bias analysis using the MINORS criteria.

|  | Methodological items for non-randomized studies | Spies  2015 | Holländer  2016 | Spies  2017 a | Spies  2017 b | Lorenz  2019 | Spies  2019 | Kohal  2020 | Kunavisarut  2020 | Sala  2022 | Rutkowski  2022 | Kohal  2020 |
| --- | --- | --- | --- | --- | --- | --- | --- | --- | --- | --- | --- | --- |
| 1 | A clearly stated aim | 2 | 2 | 2 | 2 | 2 | 2 | 2 | 2 | 2 | 2 | 2 |
| 2 | Inclusion of consecutive patients: a | 2 | 2 | 2 | 2 | 2 | 2 | 2 | 2 | 2 | 2 | 2 |
| 3 | Prospective collection of data | 2 | 2 | 2 | 2 | 2 | 2 | 2 | 2 | 0 | 0 | 2 |
| 4 | Endpoints appropriate to the aim of the study | 2 | 2 | 2 | 2 | 2 | 2 | 2 | 2 | 2 | 2 | 2 |
| 5 | Unbiased assessment of the study endpoint | 0 | 2 | 1 | 1 | 2 | 2 | 1 | 2 | 1 | 1 | 1 |
| 6 | Follow-up period appropriate to the aim of the study | 2 | 2 | 2 | 2 | 2 | 2 | 2 | 2 | 2 | 2 | 2 |
| 7 | Loss to follow up less than 5% | 2 | 2 | 2 | 1 | 0 | 0 | 1 | 2 | 2 | 2 | 2 |
| 8 | Prospective calculation of the study size | 0 | 0 | 2 | 2 | 0 | 2 | 0 | 0 | 0 | 0 | 0 |
| 9 | An adequate control group | - | - | - | - | - | - | - | - | - | - | - |
| 10 | Contemporary groups: | - | - | - | - | - | - | - | - | - | - | - |
| 11 | Baseline equivalence of groups | - | - | - | - | - | - | - | - | - | - | - |
| 12 | Adequate statistical analyses: | - | - | - | - | - | - | - | - | - | - | - |
| # | Overall score | 12 | 14 | 15 | 14 | 12 | 14 | 12 | 14 | 11 | 11 | 13 |
